# An Analysis of Phenotype and Genotype in a Large Cohort of Chinese Children with Angelman Syndrome

**DOI:** 10.3390/genes13081447

**Published:** 2022-08-14

**Authors:** Xiaonan Du, Ji Wang, Shuang Li, Yu Ma, Tianqi Wang, Bingbing Wu, Yuanfeng Zhou, Lifei Yu, Yi Wang

**Affiliations:** 1 Division of Neurology, National Children’s Medical Center, Children’s Hospital of Fudan University, No. 399 Wanyuan Road, Shanghai 201102, China; 2 Center for Molecular Medicine, National Children’s Medical Center, Children’s Hospital of Fudan University, No. 399 Wanyuan Road, Shanghai 201102, China; 3 Department of Neuroelectrophysiology, National Children’s Medical Center, Children’s Hospital of Fudan University, No. 399 Wanyuan Road, Shanghai 201102, China; 4 Division of Hosptial Administration, Shanghai Medical College, Fudan University, 138 Yixueyuan Road, Shanghai 200032, China

**Keywords:** Angelman syndrome, phenotype and genotype, Chinese children, 15q deletion, *UBE3A*

## Abstract

Angelman syndrome (AS) is a neurodevelopmental genetic disorder, but there has been limited analysis of a large cohort of Chinese children with Angelman syndrome. This study aims to assess the phenotype and genotype of Chinese children with Angelman syndrome. We retrospectively analyzed data through a detailed online survey combined with an on-site study. Furthermore, phenotype analysis stratified by deletion and non-deletion groups was carried out. The responses of family members of 695 individuals with AS revealed that 577 patients (83.02%) had maternal deletions, 65 patients (9.35%) carried *UBE3A* mutations, 31 (4.46%) patients had UPD15pat (one patient with UPD15pat constituted by a mosaic), 10 patients (1.44%) had imprinting defects and 12 (1.58%) patients only showed abnormal methylation without further detection. We identified 50 different pathogenic variants in this cohort, although 18 of these variants were unreported. Recurrent variant c.2507_2510del (p.K836Rfs*4) was found in 7 patients. In the deletion group, patients were diagnosed at an earlier age, had a more severe clinical phenotype, a higher rate of epilepsy with more multiple seizure types, and more frequently combined medication. Strabismus and sleep disturbances were both common in deletion and non-deletion groups. The top three resources invested in caring for AS children are: daily involvement in patient care, rehabilitation cost, and anti-epileptic treatment. Our study showed the genetic composition of Chinese children with 83.02% of maternal deletions, and the mutation spectrum for *UBE3A* variants was expanded. Developmental outcomes are associated with genotype, and this was confirmed by deletion patients having a worse clinical phenotype and complex epilepsy.

## 1. Introduction

Angelman syndrome (AS/OMIM #105830) is a neurodevelopmental genetic disorder first described by Dr. Harry Angelman in 1965 [1]. AS is a rare disorder; the incidence of AS ranges between 1:10,000 and 1:62,000 [2,3,4], and the incidence of AS in Hong Kong is approximately 1 per 22,305 live births [5]. AS is characterized by severe intellectual disability, lack of speech, a happy disposition, ataxia, difficulties with motor control and planning, significant sleep difficulties, epilepsy, and a distinct behavioral profile [6].

The underlying etiology of AS is the lack of expression of the maternally inherited *UBE3A* gene in the 15q11-13 imprinted region chromosome 15q11-13 [7,8]. *UBE3A* encodes a HECT domain E3 ubiquitin ligase that targets substrate proteins, transferring the ubiquitin to the proteins targeted for degradation by the ubiquitin-proteasome system [9,10]. The most common subtype that occurs in approximately 65–75% of affected individuals is the “deletion subtype”, followed by *UBE3A* mutations (8–11%), paternal uniparental disomy for chromosome15 (UPD 15pat, 3–7%), and imprinting defects (3%) [11]. The clinical spectrum and molecular study of AS have only been reported in a small sample of Chinese individuals. In a study regarding the genetic composition among Chinese AS patients in a small cohort of 49 patients, the deletion subtype accounted for 83.7% of patients, and paternal uniparental disomy, imprinting defects, and *UBE3A* gene mutations each accounted for 4.1% (2/49) [12]. In another study in Hong Kong, 65.5% of 55 AS cases were caused by maternal microdeletion, 10.9% by paternal uniparental disomy, 3.6% by imprinting defects, and 14.5% by *UBE3A* gene mutation [5]. However, the incidence of microcephaly has been reported to be lower in the Chinese and Japanese populations compared with the Caucasian population [13]. This previous study may indicate that the genetic composition, as well as the phenotype, in AS may vary among different populations.

Although AS is a rare disorder, the clinical features are severe and lifelong, resulting in significant individual and family burdens, and presumably economic and societal burdens [14]. Until now, no studies have explored the financial or societal burden, which results in a significant economic burden.

There are currently no gene-specific treatments for AS patients; treatment and management focus on the standards of care in the management of AS [15,16]. Encouraging pre-clinical studies suggest that new treatments could be approved in the near future, especially for antisense oligonucleotides treatment and Cas9 gene therapy aimed at reactivating the silenced paternal copy of *UBE3A* by downregulating *UBE3A*-ATS [17,18,19]. Identifying significant baseline phenotypes and genotypes, their clinical needs, and burden in terms of AS has been an ongoing issue in different populations. Therefore, this retrospective study aimed to summarize the clinical and genetic findings of all molecular confirmed AS patients in Chinese children in a large sample.

## 2. Materials and Methods

### 2.1. Patient Recruitment

Approximately 1000 families of individuals with AS were contacted through the Chinese Angelman Syndrome Organization and in the neurology department of Children’s Hospital of Fudan University. The parents of AS were asked to complete a questionnaire survey online about the genetic subtypes of AS (Questionnaire S1). Patients of AS were molecular confirmed before recruitment by the qualified laboratories. The gene reports have been double-checked. The genetic subtypes included: (1) maternal deletion of chromosome 15q11–13; (2) *UBE3A* mutations (Transcript:NM 130838/130839/000462); (3) UPD15pat; and (4) imprinting defects. Patients with an alternative diagnosis after assessment and genetic investigation were excluded.

### 2.2. Study Design and Timing of Assessments

After screening and determination of eligibility, the molecular confirmed patients underwent a thorough baseline examination: including a questionnaire survey with basic demographic information and clinical records (Questionnaire S2). The clinical records included appearance, behavior, epilepsy, microcephaly, development of gross motor skills, language performance, auxiliary examinations (EEG monitoring, cranial magnetic resonance imaging (MRI)/computed tomography (CT), and metabolic screening tests), comorbidity and family history. A portion of AS patients available got an on-site interview and physical examination in the clinical division of Neurology of Children’s Hospital of Fudan University (n = 253). The medical history records in medical institutions of the patients who only have questionnaires will be reviewed. With the family’s consent, we will make a telephone inquiry to supplement the missing items. The survey was available online for 3 months, from May to August 2021.

### 2.3. Statistical Analysis

The study was designed as a retrospective analysis and the sample size was based on enrollment projections through the Chinese Angelman Syndrome Organization, rather than on statistical justifications.

The statistical program SPSS version 22.0 (SPSS Inc, Chicago, IL, USA) was used for data entry and analysis. For statistical calculation, age as the continuous variable was expressed as mean ± SD and compared by Student’s *t*-test; the percentage as categorical variables was compared by Fisher’s exact test. Two-tailed *p*-values were also computed. Relative risk and 95% confidence interval were calculated for outcome variables. A *p*-value of <0.05 was considered statistically significant.

## 3. Results

### 3.1. Patients and Genetic Studies

There were valid responses from family members of 695 individuals with AS from questionnaires S1 and S2, representing a response rate of roughly 70%, and an average age of 6.34 ± 2.94 (1.24–23.16 years) at the time of the survey. Two patients were older than 18. No significant difference between genders was noted.

The underlying genetic mechanisms are summarized in Table 1, revealing that 577 patients (83.02%) had maternal deletions, 65 patients (9.35%) carried *UBE3A* mutations, 31 (4.46%) patients had UPD15pat (one patient with UPD15pat constituted by a mosaic) and 10 patients (1.44%) had maternal imprinting defects; 12 patients only showed the abnormal methylation by MLPA-MS without further analysis to differentiate whether those AS were due to UPD15pat or imprinting defects. The mean age at diagnosis among different genetic subtypes was summarized in Table 1, as well as stratified by age (Supplementary Materials Figure S1). In the deletion type, the mean diagnosed age was 24.19 ± 17.30 months. We divided the children into different age groups, and found the molecular diagnosis age was getting younger and the rate of early diagnosis is improving.

Case S100 was a 7-year-old boy with mild-moderate developmental delays, mild hand tremors, and ataxia, with no seizures, dysmorphic features, or obesity. The CMA identified mosaic for genome-wide paternal UPD-spanning chromosome 15, suggestive of uniparental isodisomy 15, and the allele difference showed the proportions of mosaic degrees in peripheral blood DNA to be 40%. These followed MLPA-MS and showed abnormal methylation, confirming the diagnosis to be AS.

### 3.2. Mutation Spectrum for UBE3A Variants in Angelman Syndrome

We identified 50 different pathogenic variants in 59 sporadic cases and in pedigrees of three families of our cohort (Supplementary Materials Table S1). 18 of these variants were unreported. 19 were proved to be familial, while 46 others were de novo.

**Figure 1:**
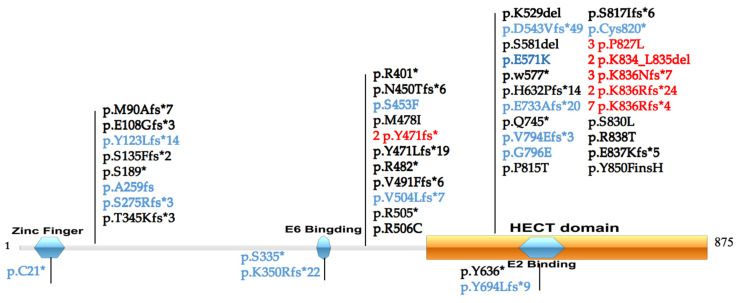
Variants in *UBE3A* protein relative to functional domains (NM_130838). Functional domains of *UBE3A* collected from the Conserved Domain Database (CDD) included Zinc finger (21–60), E6 Binding (378–396), HECT domain (518–875) and E2 Binding (635–694). The variants identified in this study are highlighted in blue. The recurrence variants are highlighted in red. * Stop codons.

31 (62%) of these variants were a result of small insertions and deletions, and 19 (38%) are single nucleotide substitutions. Three splice site and translation error variants at the consensus intronic splice sites are c.2364-1G>A, c.2438+2T>C and c.2625-*6del, which are predicted to affect the mRNA processing. The distribution of *UBE3A* variants across the functional domains are shown in Figure 1.

The recurrent variants were c.2507_2510del (p.K836Rfs*4) in 7 patients, c.2503_2507dup (p.K836Nfs*7) and c.2480C>T (p.P827L) in 3 patients, as well as c.2503_2508del (p.K834_L835del), c.2507_2508del (p.K836Rfs*24) and c.1412_1416del (p.Y471fs*) in 2 patients. These variants have been found in most patients series.

### 3.3. Schematic Representation of Clinical Manifestations of Angelman Syndrome

The maternal deletions accounted for up to 83.02%, defined as the deletion group, while those with *UBE3A* mutation, UPD15pat, and imprinting defects were combined together as the non-deletion group (16.98%). Twelve patients only showed abnormal methylation by MLPA-MS without deletion identified. This result confirms the diagnosis of AS, and the molecular cause of AS may be due to paternal UPD or an imprinting defect. They were added to the nondeletion group. The clinical features of AS in this study are summarized in Table 2. In the deletion and nondeletion groups, the percentages of microcephaly (2 SD of the normal occipitofrontal circumference (OFC) were 51.13% vs. 40.68%, respectively; *p* < 0.05), whereas strabismus (54.07% vs. 53.39%, respectively) was similar. Obesity was higher in the non-deletion group.

Among our cohort, cognition impairment was obvious except for the mosaic homozygous of UPD. A total of 119 children were evaluated using the Chinese version of the Griffith Mental Development Scale (GMDS-C), the median GQ score of the GMDS was 29.6 points (95% confidence interval, 28.6–33.25) (our published paper [20]). Speech impairment was also very obvious. When comparing the two groups, the vocal language ability was relatively better in the non-deletion group, members of which could verbalize short sentences.

Patients in the deletion group learned to sit by themselves at an average age of 14.13 ± 9.29 months, and walk by themselves at 40.9 ± 15.52 months, much later than those in the non-deletion group who learned to sit at an average age of 11.3 ± 6.7 months and walk at 34.19 ± 13.31 months (*p* < 0.01). For the patients older than 5 in the deletion group, 35.81% were unable to walk by themselves. Sleep disturbances were common both in the deletion and non-deletion groups (89.08% vs. 83.90%). Behavioral profiles are described in Table 2. In the deletion group, abnormal food-related behaviors were more serious, while the non-deletion groups had more autonomous behaviors.

Epilepsy was typical and common in AS patients, and the deletion group had a higher rate of epilepsy (86.48%) than the non-deletion group (46.61%). The first seizure age was significantly younger in the deletion group at 23.61 ± 13.26 months, while in the non-deletion group the seizure onset age was 29.64 ± 17.36 months (*p* < 0.05). A total of 14.98% of patients reported experiencing convulsive status epilepticus or status epilepticus with atypical absence/myoclonic which was recorded by video electrooculography (VEEG) (as defined as seizures lasting over 30 min). Based on the seizure history described by caregivers, more multiple seizure types (>2-types) were observed in 72% of subjects of the deletion group, while 52.73% in the non-deletion group. Fever was the most common trigger to induce seizures. Fever-induced seizures occurred in 67.73% of AS with deletion and 60% in the non-deletion group. For those with maternal deletions, combined medication was more frequently used, including combining >2 antiseizure drugs (ASDs) (57.29% n = 473). However, 56.26% (n = 48) of those in the non-deletion group were treated by monotherapy. Valproate (VPA) was the most common initial ASD, followed by Levetiracetam (LEV) and Benzodiazepines (BDZs). VPA plus with LEV was the most common two-drug combination, and VPA, LEV, and BDZs were the most common three-drug combination. In the deletion group, eight subjects (1.6%) tried the ketogenic diet, and two patients used vagus nerve stimulation.

### 3.4. Clinical Needs and Burden in Angelman Syndrome

Given the severity of the AS phenotype, the burden on caregivers was surveyed. Firstly, in this cohort, the top three resources invested in caring for AS children are daily involvement in patient care, rehabilitation costs, and anti-seizure treatment. A total of 49.75% of families needed at least one parent as a full-time caregiver for their AS child.

For children diagnosed with AS, the main reasons for seeking medical advice were seizures (51.4%), sleep disturbances (16.47%), pneumonia (6.75%), strabismus (4.12%), accidental injuries (2.32%), and teeth problems (1.15%). The top three treatment needs for AS families were speech/communication ability, reducing seizures, and cognitive improvement.

## 4. Discussion

In our study, there were 695 molecular confirmed AS cases, which is the largest study group with Chinese children. Secondly, the mutation spectrum for *UBE3A* variants were expanded. Lastly, further phenotype analysis stratified by deletion and non-deletion groups was conducted. In the deletion group, patients were diagnosed at an earlier age, and showed more severe clinical phenotype and complex epilepsy. Besides, our study has confirmed and replicated the feature of AS in other studies.

In Western populations, the most common deletion subtype is in approximately 65–75% of affected individuals. Our cohort revealed that 577 patients (83.02%) had maternal deletions. The prevalence of different molecular mechanisms differed from previous reports because of a higher rate of maternal deletions. Firstly, we found the molecular diagnosis age to be getting younger and the rate of early diagnosis was improving, especially in the deletion type. In addition patients with deletion showed more significant facial features and higher epilepsy prevalence, which can be diagnosed easier. Secondly, the most commercially available MS-MLPA kit was typically the first test ordered. If sequence analysis of *UBE3A* was not ordered, the diagnosis of some patients will be missed. Thirdly, all patients were molecular confirmed before recruitment and no suspected patients were recruited. Therefore, we speculated that the percent composition of deletion would be exaggerated to some degree. Conversely, this reminded us to increase awareness of patients with non-deletion genetic subtypes, especially in the older population.

Loss of functional *UBE3A* gene expression is the principal reason for and cause of Angelman syndrome. Mutations in the *UBE3A* gene causing AS have been found in approximately 10% of the cases. Multiple nucleotide deletions or insertions can be recurrent. The recurrent variants were c.2507_2510del (p.K836Rfs*4) in 7 patients, c.2503_2507dup (p.K836Nfs*7) and c.2480C>T (p.P827L) in 3 patients, as well as c.2503_2508del (p.K834_L835del),c.2507_2508del (p.K836Rfs*24) and c.1412_1416del (p.Y471fs*) in 2 patients.These variants have been found in most patient series [21,22,23]. This high proportion of these points may suggest recurrent hot points.

There was one patient who harbored mosaicism of UPD15pat in this cohort. Previous studies have reported that patients can have milder or atypical AS with mosaic imprinting defects [24,25] and our patient also showed milder AS features. Our study retrospectively reviewed the genotype of AS patients in China and expand the spectrum of genetic findings.

Children with AS have a distinct developmental and behavioral profile. Developmental delays (moderate to severe), absent speech, and abnormal EEG are consistent in different ages among different genetic subtypes. J.-L. Bai reported that the frequency of microcephaly was much lower (36.7%) in a Chinese cohort (n = 44); however, in our larger sample of patients, 50.26% had microcephaly (2 SD of normal occipitofrontal circumference), which was consistent with reports in the western population. Sleep problems are present in up to 80% of individuals with AS, including problems with settling and insomnia, awakenings during the night, early awakening, and sleep tends to be more fragmented [26]. Sleep disturbances were both common in deletions and non-deletion groups in our cohort. Poor sleep quality may impact the regulation of behavior and worsen seizures, which should be received attention and be managed. Sleeping difficulties decrease with age, although many adolescents and adults continue to have disordered sleep.

For these children diagnosed with AS, the first main reason for seeking medical advice was seizures. Epilepsy is very common in AS, especially for the deletion group, who had a higher rate of epilepsy, an earlier first seizure age, more multiple seizure types, and more frequently combined medication; these data were consistent with the previous study of 461 family members of individuals with AS, which was also based on questionnaire assessment [27]. Notably, the seizure types were reported based on the seizure history described by caregivers, and long-term video EEG recording helpd determine if a behavioral episode is a seizure or a non-epileptic event. For up to 1/3 of individuals, the first seizure will occur in the setting of a febrile illness. There are no comparative trials of the various antiseizure drugs; the consensus recommendation is to treat with clobazam or levetiracetam as first-line therapy and to consider dietary intervention, including a ketogenic diet and low glycemic index treatment. Besides, the use of valproic acid caused a high rate of motor sides effects in the previous study [28,29]. While in our study, VPA was the most common initial ASD and common drug combination. The use of the ketogenic diet was low in this cohort. The result directly mirrors drugs prescribed in the real world in the Chinese children, and caregivers should be educated on seizure management and pay attention to the exacerbations related to ASDs. The further characterization of epilepsy in AS will hopefully lead to a better understanding of the pathogenesis of epilepsy in this population and better approaches to effectively treating epilepsy in AS. For the deletion group, the GABRB3 maps to the region of 15q involved in AS could partially explain the deletion patients having worse epilepsy than other patients in the non-deletion group [30].

It is important to note that although seizures was the initial primary reason for seeking medical care, the top clinical need for AS families was speech/communication ability. Although children may communicate through gestural communication and the electronic augmentative and alternative communication (AAC) devices [31,32], the lack of verbal communication may result in frustration for patients as well as their caregiver. The integrated quantitative MR imaging analysis demonstrated poor functional and structural connectivity and brain volume reduction in children with AS, which may explain the language dysfunction; there is a need to study more to explore the speech pathway in the brain [33,34]. However, only a few children with AS have tried AAC devices in China, which practitioners may encourage more patients to try for communication. Caregivers are at high risk for experiencing negative consequences. In our study, the top resource invested in caring for AS children was daily involvement in patient care, and 49.75% of families needed at least one parent as a full-time caregiver for AS children. The investment of time and daily involvement in patient care are difficult to measure economically, however the AS families deserve more attention for their caregiver burden.

Understanding the natural history of the disorder informs the assessment of treatment efficacy in terms of both clinical outcomes and design for novel therapies. This study is critical to increasing awareness and standards of care of AS patients to ensure optimal care, as well as providing a basis for clinical trials in the treatment of AS in the future. However, there are still some limitations in our study; firstly, all patients were molecular confirmed before recruitment, and there were no suspected patients recruited. Secondly, some clinical features of AS patients in our study were summarized retrospectively, and the recalling bias should be considered. This cohort will be followed up in order to study the natural history of AS patients.

## 5. Conclusions

This is the largest molecular and clinical study of AS in the Chinese children to date. Our study showed the genetic composition of Chinese children with 83.02% of maternal deletions. The mutation spectrum for *UBE3A* variants was expanded. Developmental outcomes are associated with genotype and confirmed by deletion patients having worse clinical phenotype and complex epilepsy.

## Figures and Tables

**Table 1:** Pattern of underlying genetic mechanisms for AS in our study.

Total Number (n = 695)	Genotypes				
	15q11.2-q13del	*UBE3A* mutation	UPD15pat	Imprinting defect	Unclear (UPD15pat/ID)
Number	577	65	31	10	12
%	83.02%	9.35%	4.46%	1.44%	1.58%
M:F	295:282	30:35	18:13	3:7	5:7
Diagnosed age (months)	24.19 ± 17.30	37.16 ± 38.89	30.12 ± 12.88	28.71 ± 13.06	38.31 ± 38.59

AS: Angelman syndrome; del: deletion; UPD15pat; paternal uniparental disomy for chromosome15; Imprinting defect: ID; M: Male; F: Female.

**Table 2:** Comparison of clinical features in AS caused by deletion versus non-deletion.

Clinical Feature		Del (N = 577) /Per%		Non-Del (N = 118) /Per%		*p*
Epilepsy		499	86.48%	55	46.61%	<0.0001 (****)
Sleep problems		514	89.08%	99	83.90%	NS
Feeding problems		480	83.19%	84	71.19%	<0.01 (**)
Speech impairment		577	100%	118	100%	NS
	No use of words	463	80.24%	60	50.85%	<0.0001 (****)
	Verbal (fewer than 2 words or word approximations)	94	16.29%	52	44.07%	<0.0001 (****)
	Short sentences	5	0.87%	6	5.08%	<0.001 (***)
Facial features	Microcephaly	295	51.13%	48	40.68%	<0.05 (*)
	Strabismus	312	54.07%	63	53.39%	NS
	Saprodontia	185	32.06%	48	40.68%	NS
	Widely spaced teeth	310	53.73%	59	50.00%	NS
	Light skin	446	77.30%	33	27.97%	<0.0001 (****)
	Obesity	22	3.81%	22	18.64%	<0.0001 (****)
	Scoliosis	101	17.50%	12	10.17%	<0.05 (*)
Behavioral features	Hypermotoric	544	94.28%	103	87.29%	<0.01 (**)
	Abnormal food-related behaviors	462	80.07%	80	67.80%	<0.001 (***)
	Fascination with crinkly items	359	62.22%	72	61.02%	NS
	Autotomy	26	4.51%	14	11.86%	<0.01 (**)
	Attraction to and fascination with water	414	71.75%	95	80.51%	NS
	Stereotyped behavior	165	28.60%	35	29.66%	NS
	Asphyxia due to foreign body	53	9.19%	14	11.86%	NS

NS: not statistically significant; Del: deletion; Non-del: non-deletion. * *p* < 0.05; ** *p* < 0.01; *** *p* < 0.001; **** *p* < 0.0001

## Data Availability

The datasets used and/or analyzed during the current study are available from the corresponding author on reasonable request.

## Supplementary Materials

The following supporting information can be downloaded at: https://www.mdpi.com/article/10.3390/genes13081447/s1, Figure S1: The mean age at diagnosis were stratified by age among different genetic subtypes; Table S1: *UBE3A* Pathogenic Variants

## References

[1] Williams C.A., Frias J.L. (1982). The Angelman (“happy puppet”) syndrome. Am. J. Med. Genet..

[2] Petersen M.B., Brondum-Nielsen K., Hansen L.K., Wulff K. (1995). Clinical, cytogenetic, and molecular diagnosis of Angelman syndrome: Estimated prevalence rate in a Danish county. Am. J. Med. Genet..

[3] Mertz L.G., Christensen R., Vogel I., Hertz J.M., Nielsen K.B., Gronskov K., Ostergaard J.R. (2013). Angelman syndrome in Denmark. birth incidence, genetic findings, and age at diagnosis. Am. J. Med. Genet. A.

[4] Thomson A.K., Glasson E.J., Bittles A.H. (2006). A long-term population-based clinical and morbidity profile of Angelman syndrome in Western Australia: 1953–2003. Disabil. Rehabil..

[5] Luk H.M., Lo I.F. (2016). Angelman syndrome in Hong Kong Chinese: A 20 years’ experience. Eur. J. Med. Genet..

[6] Williams C.A., Beaudet A.L., Clayton-Smith J., Knoll J.H., Kyllerman M., Laan L.A., Magenis R.E., Moncla A., Schinzel A.A., Summers J.A. (2006). Angelman syndrome 2005: Updated consensus for diagnostic criteria. Am. J. Med. Genet. A.

[7] Jiang Y., Lev-Lehman E., Bressler J., Tsai T.F., Beaudet A.L. (1999). Genetics of Angelman syndrome. Am. J. Hum. Genet..

[8] Maranga C., Fernandes T.G., Bekman E., da Rocha S.T. (2020). Angelman syndrome: A journey through the brain. FEBS J..

[9] Jiang Y.H., Armstrong D., Albrecht U., Atkins C.M., Noebels J.L., Eichele G., Sweatt J.D., Beaudet A.L. (1998). Mutation of the Angelman ubiquitin ligase in mice causes increased cytoplasmic p53 and deficits of contextual learning and long-term potentiation. Neuron.

[10] Lopez S.J., Laufer B.I., Beitnere U., Berg E.L., Silverman J.L., O’Geen H., Segal D.J., LaSalle J.M. (2019). Imprinting effects of UBE3A loss on synaptic gene networks and Wnt signaling pathways. Hum. Mol. Genet..

[11] Buiting K., Clayton-Smith J., Driscoll D.J., Gillessen-Kaesbach G., Kanber D., Schwinger E., Williams C., Horsthemke B. (2015). Clinical utility gene card for: Angelman Syndrome. Eur. J. Hum. Genet..

[12] Bai J.L., Qu Y.J., Jin Y.W., Wang H., Yang Y.L., Jiang Y.W., Yang X.Y., Zou L.P., Song F. (2014). Molecular and clinical characterization of Angelman syndrome in Chinese patients. Clin. Genet..

[13] Saitoh S., Harada N., Jinno Y., Hashimoto K., Imaizumi K., Kuroki Y., Fukushima Y., Sugimoto T., Renedo M., Wagstaff J. (1994). Molecular and clinical study of 61 Angelman syndrome patients. Am. J. Med. Genet..

[14] Wheeler A.C., Sacco P., Cabo R. (2017). Unmet clinical needs and burden in Angelman syndrome: A review of the literature. Orphanet. J. Rare Dis..

[15] Duis J., Nespeca M., Summers J., Bird L., Bindels-de Heus K., Valstar M.J., de Wit M.Y., Navis C., Ten Hooven-Radstaake M., van Iperen-Kolk B.M. (2022). A multidisciplinary approach and consensus statement to establish standards of care for Angelman syndrome. Mol. Genet. Genomic Med..

[16] Bonello D., Camilleri F., Calleja-Agius J. (2017). Angelman Syndrome: Identification and Management. Neonatal Netw..

[17] Wolter J.M., Mao H., Fragola G., Simon J.M., Krantz J.L., Bazick H.O., Oztemiz B., Stein J.L., Zylka M.J. (2020). Cas9 gene therapy for Angelman syndrome traps Ube3a-ATS long non-coding RNA. Nature.

[18] Markati T., Duis J., Servais L. (2021). Therapies in preclinical and clinical development for Angelman syndrome. Expert Opin. Investig. Drugs.

[19] Copping N.A., McTighe S.M., Fink K.D., Silverman J.L. (2021). Emerging Gene and Small Molecule Therapies for the Neurodevelopmental Disorder Angelman Syndrome. Neurotherapeutics.

[20] Li S., Ma Y., Wang T., Jin H., Du X., Wang Y. (2022). Epilepsy and Molecular Phenotype Affect the Neurodevelopment of Pediatric Angelman Syndrome Patients in China. Front. Psychiatry.

[21] Camprubi C., Guitart M., Gabau E., Coll M.D., Villatoro S., Oltra S., Rosello M., Ferrer I., Monfort S., Orellana C. (2009). Novel UBE3A mutations causing Angelman syndrome: Different parental origin for single nucleotide changes and multiple nucleotide deletions or insertions. Am. J. Med. Genet. A.

[22] Ferrarini A., Xumerle L., Griggio F., Garonzi M., Cantaloni C., Centomo C., Vargas S.M., Descombes P., Marquis J., Collino S. (2015). The Use of Non-Variant Sites to Improve the Clinical Assessment of Whole-Genome Sequence Data. PLoS ONE.

[23] Sadikovic B., Fernandes P., Zhang V.W., Ward P.A., Miloslavskaya I., Rhead W., Rosenbaum R., Gin R., Roa B., Fang P. (2014). Mutation Update for UBE3A variants in Angelman syndrome. Hum. Mutat..

[24] Lawson-Yuen A., Wu B.L., Lip V., Sahoo T., Kimonis V. (2006). Atypical cases of Angelman syndrome. Am. J. Med. Genet. A.

[25] Aypar U., Hoppman N.L., Thorland E.C., Dawson D.B. (2016). Patients with mosaic methylation patterns of the Prader-Willi/Angelman Syndrome critical region exhibit AS-like phenotypes with some PWS features. Mol. Cytogenet..

[26] Miano S., Bruni O., Leuzzi V., Elia M., Verrillo E., Ferri R. (2004). Sleep polygraphy in Angelman syndrome. Clin. Neurophysiol..

[27] Thibert R.L., Conant K.D., Braun E.K., Bruno P., Said R.R., Nespeca M.P., Thiele E.A. (2009). Epilepsy in Angelman syndrome: A questionnaire-based assessment of the natural history and current treatment options. Epilepsia.

[28] Shaaya E.A., Grocott O.R., Laing O., Thibert R.L. (2016). Seizure treatment in Angelman syndrome: A case series from the Angelman Syndrome Clinic at Massachusetts General Hospital. Epilepsy Behav..

[29] Grocott O.R., Herrington K.S., Pfeifer H.H., Thiele E.A., Thibert R.L. (2017). Low glycemic index treatment for seizure control in Angelman syndrome: A case series from the Center for Dietary Therapy of Epilepsy at the Massachusetts General Hospital. Epilepsy Behav..

[30] Wagstaff J., Knoll J.H., Fleming J., Kirkness E.F., Martin-Gallardo A., Greenberg F., Graham J.M., Menninger J., Ward D., Venter J.C. (1991). Localization of the gene encoding the GABAA receptor β3 subunit to the Angelman/Prader-Willi region of human chromosome 15. Am. J. Hum. Genet..

[31] Quinn E.D., Rowland C. (2017). Exploring Expressive Communication Skills in a Cross-Sectional Sample of Children and Young Adults with Angelman Syndrome. Am. J. Speech Lang Pathol..

[32] Sadhwani A., Wheeler A., Gwaltney A., Peters S.U., Barbieri-Welge R.L., Horowitz L.T., Noll L.M., Hundley R.J., Bird L.M., Tan W.H. (2021). Developmental Skills of Individuals with Angelman Syndrome Assessed Using the Bayley-III. J. Autism. Dev. Disord..

[33] Aghakhanyan G., Bonanni P., Randazzo G., Nappi S., Tessarotto F., De Martin L., Frijia F., De Marchi D., De Masi F., Kuppers B. (2016). From Cortical and Subcortical Grey Matter Abnormalities to Neurobehavioral Phenotype of Angelman Syndrome: A Voxel-Based Morphometry Study. PLoS ONE.

[34] Yoon H.M., Jo Y., Shim W.H., Lee J.S., Ko T.S., Koo J.H., Yum M.S. (2020). Disrupted Functional and Structural Connectivity in Angelman Syndrome. AJNR Am. J. Neuroradiol..

