# Supplementary material for: An Analysis of Phenotype and Genotype in a Large Cohort of Chinese Children with Angelman Syndrome
Source: Genes (Basel). 2022 Aug 14;13(8):1447. doi: 10.3390/genes13081447 (PMC9408022; doi:10.3390/genes13081447)
Supplement: Supplementary file 1 [file genes-13-01447-s001.zip › genes-1850375-supplementary Figure S1.pdf]

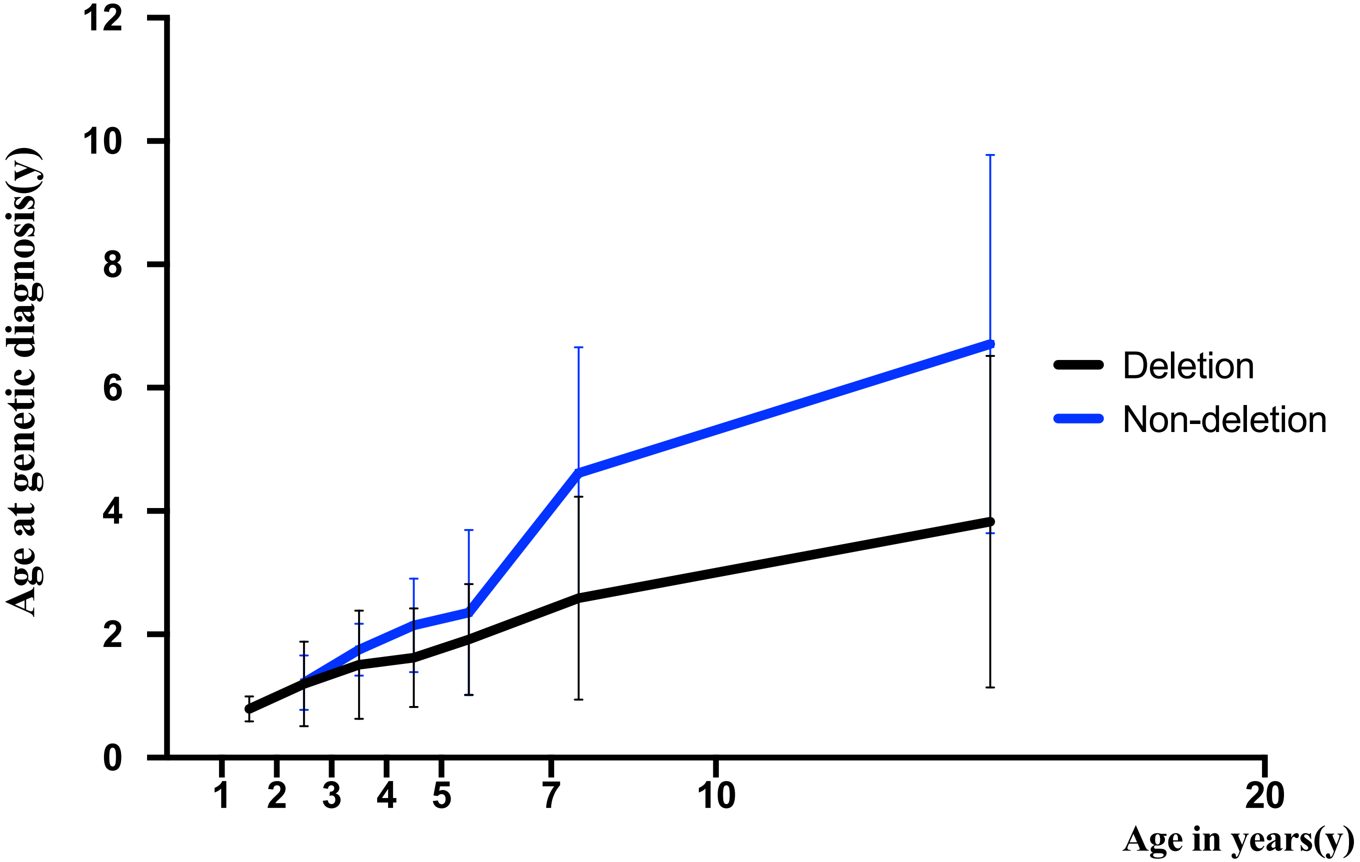

Figure S1. The mean age at diagnosis were stratified by age among different genetic subtypes

Y: Age at genetic diagnosis（y）

X: Current Age in years（y）.
